# Comparative Analysis of Tumor-Associated microRNAs and Tetraspanines from Exosomes of Plasma and Ascitic Fluids of Ovarian Cancer Patients

**DOI:** 10.3390/ijms24010464

**Published:** 2022-12-27

**Authors:** Natalia Yunusova, Ekaterina Dzhugashvili, Alena Yalovaya, Larisa Kolomiets, Aleksei Shefer, Alina Grigor’eva, Alexey Tupikin, Irina Kondakova, Svetlana Tamkovich

**Affiliations:** 1 Cancer Research Institute, Tomsk National Research Medical Center, Russian Academy of Sciences, 634009 Tomsk, Russia; 2 Department of Biochemistry and Molecular Biology, Faculty of Medicine and Biology, Siberian State Medical University, 634050 Tomsk, Russia; 3 V. Zelman Institute for Medicine and Psychology, Novosibirsk State University, 630090 Novosibirsk, Russia; 4 Institute of Chemical Biology and Fundamental Medicine, Siberian Branch of Russian Academy of Sciences, 630090 Novosibirsk, Russia

**Keywords:** exosomes, tumor-specific microRNAs, miR-24-3p, miR-101, CD151, Tspan8, ovarian cancer

## Abstract

Ovarian cancer (OC) is one of the most common and fatal types of gynecological cancer. In the early phase of OC detection, the current treatment and diagnostic methods are not efficient and sensitive enough. Therefore, it is crucial to explore the mechanisms of OC metastasis and discover valuable factors for early diagnosis of female cancers and novel therapeutic strategies for metastasis. Exosomes are known to be involved in the development, migration, and invasion of cancer cells, and their cargo could be useful for the non-invasive biopsy development. CD151- and Tspan8-positive exosomes are known to support the degradation of the extracellular matrix, and are involved in stroma remodeling, angiogenesis and cell motility, as well as the association of miR-24 and miR-101 with these processes. The objective of this study was to explore the relationship of these components of exosomal cargo, in patients with OC, to clarify the clinical significance of these markers in liquid biopsies. The levels of tetraspanins Tspan8+ and CD151+ exosomes were significantly higher in plasma exosomes of OC patients compared with healthy females (HFs). The relative levels of miR-24 and miR-101 in plasma exosomes of HFs were significantly higher than in plasma exosomes of OC patients, while the levels of these microRNAs in exosomes from plasma and ascites of ill females showed no difference. Our study revealed a strong direct correlation between the change in the ascites exosomes CD151+Tspan8+ subpopulation level and the expression levels of the ascites (R = 0.81, *p* < 0.05) and plasma exosomal miR-24 (R = 0.74, *p* < 0.05) in OC patients, which confirms the assumption that exosomal cargo act synergistically to increase cellular motility, affecting cellular processes and signaling. Bioinformatics analysis confirmed the involvement of CD151 and Tspan8 tetraspanins and genes controlled by miR-24-3p and miR-101 in signaling pathways, which are crucial for carcinogenesis, demonstrating that these tetraspanins and microRNAs are potential biomarkers for OC screening, and predictors of poor clinicopathological behavior in tumors.

## 1. Introduction

The frequency of peritoneal dissemination in primary ovarian cancer (OC), taking into account its late diagnosis, reaches high numbers and is comparable to the proportion of diagnosed advanced disease, which actually limits the effectiveness of surgical and chemotherapeutic methods of tumor treatment [1]. Ascites contains both biologically active molecules (growth factors, cytokines, proteins, metabolites, etc.) and extracellular vesicles (EVs) of tumor origin, including exosomes [2]. Cancer exosomes can both locally affect nearby cells or distantly affect cells, spreading to the tissues through blood and lymph. It was found that ascitic fluid of OC patients contains a higher concentration of exosomes than blood plasma [3]. According to different published sources, it is known that 1 mL of blood contains 5–30 × 10^7^ exosomes in healthy donors and 13–154 × 10^7^ exosomes in OC patients [3,4]. Theoretically, each exosome contains lipids, no more than 100 proteins, and no more than 300–1000 non-coding RNA [5], wherein the share of exosomal DNA does not exceed 0.025% healthy female (HF) plasma DNA [6]. According to the information, we are able to assume that these biologically active molecules (proteins and nucleic acids) of exosomal cargo should affect target cells in synergy.

Proteases play an important role in the functional activity of the exosomes. Tetraspanin-associated proteases of exosomes include a disintegrin and metalloproteinases (ADAMs), matrix metalloproteinases (MMPs), as well as their extracellular matrix metalloproteinase inducer (EMMPRIN) [7]. In previous works we found an association between the combined overexpression of proangiogenic MMP2/MMP9 on the surface of plasma/ascitic exosomes and low-volume ascites in patients with advanced OC [8]. In addition, the expression of ADAM10/ADAM17 on the surface of CD9-positive plasma exosomes in these patients was associated with the index of peritoneal carcinomatosis [8]. However, the representation and role of tetraspanins Tspan8 and CD151 in the cargo of plasma exosomes and ascitic exosomes in OC patients have not yet been studied.

The main functions of CD151 are to maintain the integrity of epithelial cells, platelet aggregation, regulation of membrane fusion, cell motility, and participation in angiogenesis and tumor metastasis [9]. CD151 is mostly expressed in endothelial cells and platelets, and is overexpressed in malignant tumors (prostate cancer, colorectal cancer, endometrial cancer, and non-small cell lung cancer) [9]. Furthermore, it was shown that CD151 is essential for OC cell survival through a ZEB-dependent mechanism and supports tumor development, so high CD151 expression in tissue is prognostic of poor clinical outcome [10]. A significant decrease in invasion and metastasis of OC was revealed with the use of anti-CD151 antibodies [11]. Since CD151 is located intracellularly in endosomal and lysosomal vesicles, it can be released from cells as a part of the exosomal cargo [12].

Tspan8 is a member of the tetraspanin family that also forms tetraspanin networks with various membrane proteins. In colorectal cancer, Tspan8 appears to have a proinvasive potential, interacting with α6β4-integrin, protein kinase C, E-cadherin, claudin-7, EpCAM, and CD44 [13]. Using an xenogenic mouse model, it has been shown that specific antibodies to Tspan8 reduce cell motility and block tumor angiogenesis in vivo [14]. It was found that around 52% OC patients have a high expression of TSPAN8 in tissue, correlated with poor survival. As for CD151, antibodies against Tspan8 reduce the frequency of OC metastasis [15].

In comparison with free-circulating miRNAs in biological fluids, miRNAs in exosomes are more stable due to the phospholipid bilayer surrounding the vesicles, which protects nucleic acids from hydrolysis by nucleases [16,17]. The principles of sorting non-coding RNAs into exosomes are not clear yet, but the list of tumor-associated exosomal miRNAs is expanding every year. Using various cellular models and tumor tissues, miRNA-101 and -24-3p have been shown to participate in epithelial-mesenchymal transition (EMT), inhibit apoptosis, stimulate proliferation and angiogenesis, and participate in cell migration and metastasis [18,19,20]. However, the representation and role of these microRNAs in exosomes of OC patients remains largely unknown.

Taking into account that CD151- and Tspan8-positive exosomes support the degradation of the extracellular matrix, are involved in stroma remodeling, angiogenesis and cell motility [9,13,21], as well as the association of miR-24 and miR-101 with these the same processes, it seems relevant to study the relationship of these components of exosomal cargo in patients with OC to clarify the clinical significance of these markers in liquid biopsies.

## 2. Results

### 2.1. Characterization of Exosomes

**Figure 1:**
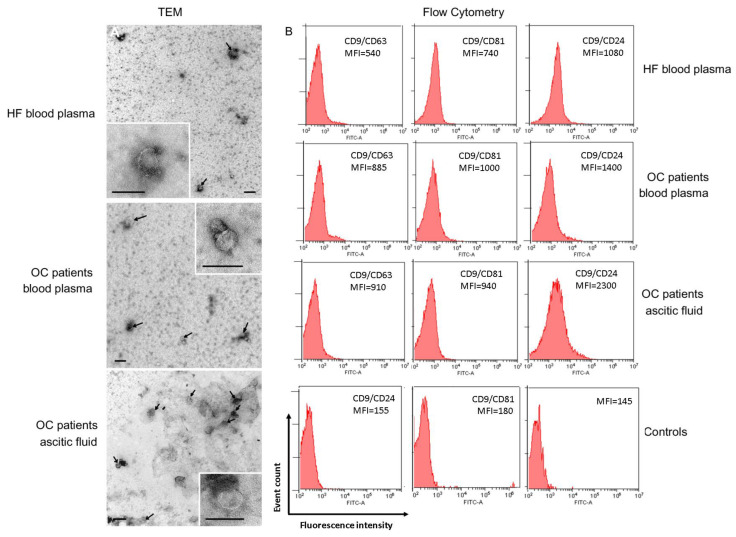
Identification of isolated exosomes. (**A**) TEM demonstrated the presence of exosome vesicles with typical cup-shape morphology (size bar 100 nm) and absence vesicles more than 100 nm. Arrows indicate exosomes, exosomes are on the insets. (**B**) Expression of CD63, CD81, and CD24 on CD9-positive exosomes of blood plasma of HFs, blood plasma, and ascites of OC patients. For flow cytometry representative median fluorescence intensity (MFI) are shown (see Table S1 for details). Each study is done in triplets. For isotype controls (histograms in the center and on the left), CD9-labeled beads-exosome complexes were incubated with FITC mouse IgG1, k Isotype control (Cat N555748, BD, Heidelberg, Germany) or FITC mouse IgG2a, k Isotype control (Cat N553456, BD, Heidelberg, Germany). For the negative control (histogram on the right), latex particles labeled with CD9 antibodies were incubated with FITC labeled antibodies anti-CD63, anti-CD81, and anti-CD24 antibody. One of the representative negative controls is shown.

The morphology of exosomes from blood plasma and ascites was examined by transmission electron microscopy (TEM) (Figure 1A). 

In samples of exosomes isolated from the blood plasma of HFs and OC patients, and ascites of OC patients, the presence of clearly structured cup-shaped vesicles of low electron density with a preserved membrane of no more than 100 nm was revealed. It has been shown that the morphology of the isolated vesicles does not differ for exosome preparations from plasma of HFs and OC patients, and from ascites of OC patients, which confirms previously published data [3,4].

To demonstrate that purified EVs were exosomes, we immobilized them onto latex beads covered with CD9 antibodies and analyzed the presence of exosomal markers CD24, CD63, and CD81 on the EV surface. Detection of these markers clearly indicated that the isolated EVs from blood plasma and ascites were exosomes indeed (Figure 1B).

Expression of CD24, CD63, and CD81 were higher in OC patient exosomes than in HF exosomes, however, the statistically significant differences were revealed in the expression of CD24 on CD9-positive exosomes (Figure 1B, Table S1), both between HF plasma exosomes and OC patient plasma exosomes, and plasma exosomes and ascite exosomes of ill patients (*p* < 0.05).

### 2.2. Distribution of CD151 and Tspan8 on the Surface of Exosomes in HFs and OC Patients

**Figure 2:**
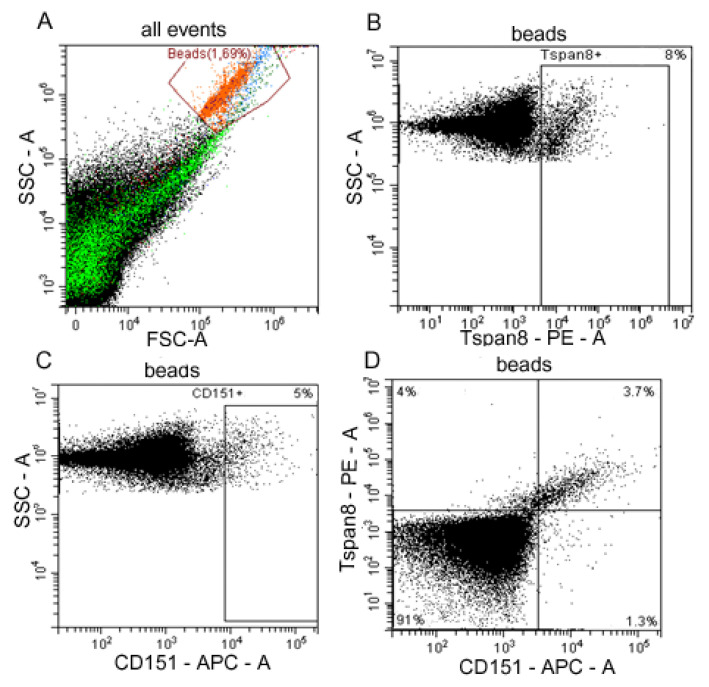
Flow cytometry analysis of plasma exosome subpopulations. (**A**) Forward scatter area (FSC-A) versus side scatter area (SSC-A) dot plot representing exosomes samples adsorbed on aldehyde/sulphate latex beads labeled anti-CD9 antibody (red), debris is marked in green. (**B**) Tspan8-positive and (**C**) CD151-positive plasma exosomes populations in OC patients. (**D**) Double labeling Tspan8 versus CD151 of OC patients’ ascites exosomes. Quantitative data on the expression of tetraspanins Tspan8 and CD151 on the surface of CD9-positive exosomes from blood plasma of HFs and blood plasma and ascites of OC patients is represented in Table 1.

We used anti-CD9 coated latex beads for the detection of CD151/Tspan8 subpopulations of exosomes in plasma of HFs and OC patients and ascites of ill females (Figure 2).

Tetraspanin compositions at the surface of the CD9-positive exosomes in HFs and OC patients are presented in Table 1. The levels of CD151+, Tspan8+, and CD151+/Tspan8+ on the surface of CD9-positive exosomes in plasma of HFs were significantly lower than in OC patients (*p* < 0.05 for each case), wherein in both cases the Tspan8+ level was three times higher than the CD151+ level (*p* < 0.05). Comparative analysis of levels of CD151+, Tspan8+, and CD151+/Tspan8+ on the surface of CD9-positive exosomes from plasma and ascitic fluids of OC patients did not reveal any differences in the content of all three subpopulations.

### 2.3. Expression of miR-24-3p and miR-101 in Exosomes from Plasma and Ascites of OC Patients

Based on the literature, two promising tumor-associated microRNAs -24-3p and -101 were selected. It has been shown in various cell models and clinical tumors that these microRNAs are involved in EMT, inhibit apoptosis, stimulate proliferation and angiogenesis, and participate in cell migration and metastasis.

**Figure 3:**
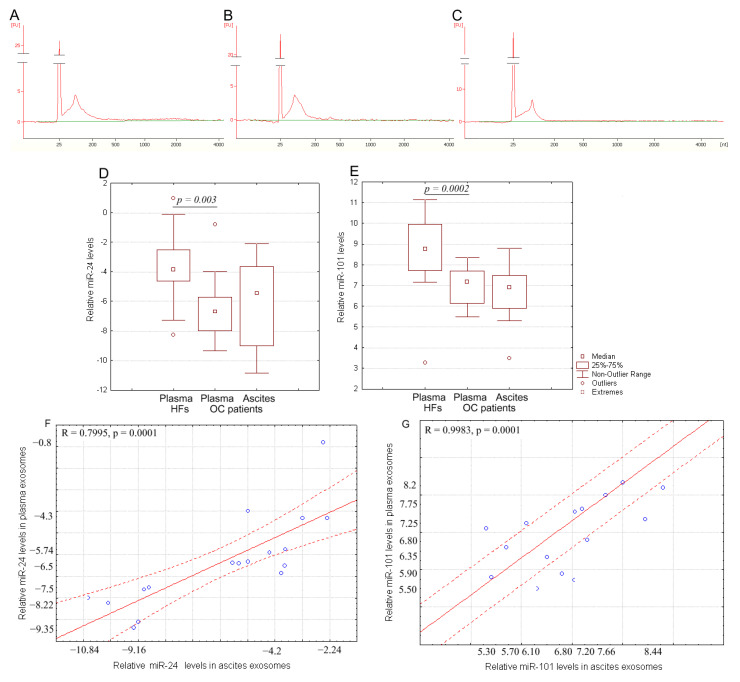
RNA content in exosomes. Size distribution of RNA extracted from pooled samples: (**A**) plasma exosomes of HFs, (**B**) plasma exosomes of OC patients, and (**C**) ascites exosomes of OC patients. The data from Agilent 2100 Bioanalyzer with 25 nt RNA fragment as an internal standard are shown. Quantification of exosomal miR-24-3p (**D**) and miR-101 (**E**) from the blood plasma of HFs and from the blood plasma and ascites fluid of OC patients. Tukey box plots of exosomal miRNAs. The statistically significant *p*-values are indicated. Correlation of miR-24-3p level (**F**) and miR-101 level (**G**) in blood plasma exosomes with ascites exosomes in OC patients. Solid red line—trend line, dotted red line—95% confidence interval.

Bioanalyzer trace analysis revealed abundant miRNA presence in the exosomes isolated from the plasma and ascites samples (Figure 3A–C). The diagnostic potential of exosomal RNA was tested by stem-loop qRT-TaqMan PCR of miR-101 and miR-24-3p, which were all down-regulated in highly metastatic human carcinoma patient tissues including OC [18]. Since the miR-16-5p expression was stable and reproducible, it was chosen as an endogenous control to normalize the miRNA expression [22,23,24]. Indeed, there was no statistical difference in the level of miR-16 from the exosomal samples from HFs and OC patients (Ct 32.09 and Ct 31.89, correspondingly). For microRNAs, qRT-PCR assays with a working range of 28.15–34.35 threshold cycles (Ct) of PCR were used. Non-template controls produced no signal or were at least seven cycles away from the minimum detectable amount of specific template. All the reported data were obtained using RNA samples that produced Ct values within the working range of the systems. Spike-in control (cel-miR-39) was detected in all the samples at 25 ± 1 Ct. Data on the miRNA relative expression in samples of exosomes from of HFs and OC patients are presented in Figure 3D,E.

The miR-24-3p relative expression in plasma exosomes of OC patients was significantly lower compared to that of the HFs (*p* < 0.01) (Figure 3D), at the same time, the relative expression of this microRNA did not differ in plasma and ascites exosomes in cancer patients. A similar pattern is observed for miR-101 (*p* < 0.01) (Figure 3E). Moreover, a statistically significant strong correlation between the relative expression of tumor-associated microRNAs in plasma exosomes and in ascites exosomes was found (R = 0.7995 and R = 0.9983 for miR-24 and for miR-101, correspondingly) (Figure 3F,G).

### 2.4. Levels of miR-24-3p in Plasma and Ascites Exosomes Are Related with Expression of CD151 and Tspan8 on CD9-Positive Exosomes

**Figure 4:**
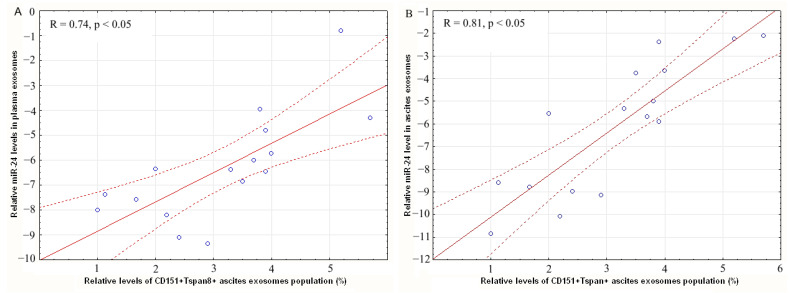
Correlation of miR-24-3p level in blood plasma exosomes (**A**) and in ascites exosomes (**B**) with level of CD151+Tspan8+ CD9+ exosomes in OC patients ascites. Solid red line–trend line, dotted red line–95% confidence interval.

The relationships between the level of CD151+Tspan8+ -ascites, CD9-positive exosomes, and relative level of miR-24-3p in plasma exosomes (R = 0.74, *p* < 0.05) and ascites exosomes (R = 0.81, *p* < 0.05) of OC patients were revealed (Figure 4). These strong (on the Chaddock scale) direct correlations between these parameters indirectly demonstrate that these tertraspanins are found in the same exosomes as miR-24-3p.

There was no correlation between miR-24-3p and miR-101 levels in plasma exosomes and in ascites exosomes with age, family history, ascites volume in OC patients, and peritoneal carcinomatosis index (PCI), but the level of miR-101 in ascites exosomes was associated with FIGO stage (*p* = 0.03) (Supplementary Materials, Tables S2 and S3).

### 2.5. Bioinformatic Analysis

It is known that microRNAs involved in carcinogenesis are not specific to a single cancer type. This is a problem of developing a highly specific diagnostic system, but it is an advantage for developing a universal marker of anticancer therapy effectiveness, especially for metastases. In any case, it is necessary to understand if the selected miRNA and gene markers are specific or universal for different oncological diseases, as well as for different therapy types. To answer these questions, further analysis using the DIANA miRPath, STRING and PANTHER databases was conducted.

**Figure 5:**
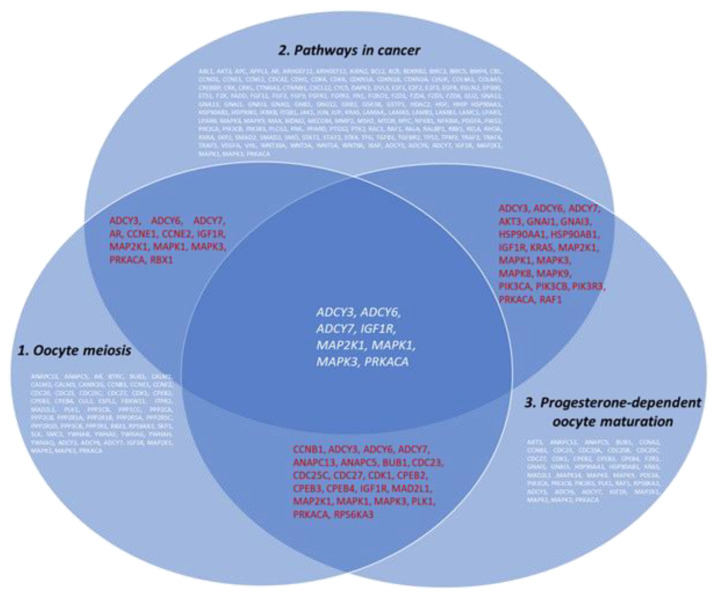
Venn-Euler diagram showing the involvement of miRNA-regulated genes in the development of OC (DIANA database).

The participation of selected miRNAs in the development of epithelial OC was analyzed using the DIANA database (http://dianalab.e-ce.uth.gr/, accessed on 30 May 2022). It was revealed that microRNA -24-3p and -101 are involved in the OC development, in particular, in oocyte meiosis and progesterone-dependent maturation of oocytes, regulating 8 genes (Figure 5).

**Figure 6:**
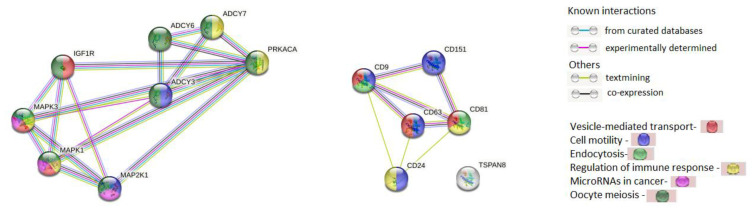
The interactions of proteins coded by genes that are regulated by miR-24 and miR-101, which are involved in oocyte meiosis, progesterone-dependent oocyte maturation and cancer pathways and exosome-associated tetraspanins (STRING Database).

The interactions of 8 proteins (ADCY3, ADCY6, ADCY7, IGF1R, MAP2K1, MAPK1, MAPK3, and PRKACA) encoded by the genes regulated by selected miRNAs involved in oocyte meiosis, progesterone-dependent oocyte maturation and the cancer pathways and 6 exosomal proteins (CD9, CD24, CD63, CD81, CD151, and TSPAN8) were analyzed using the STRING database (string-db.org). The complicated and closed network organized by these genes and the interactions between them are presented in Figure 6. It was found that genes regulated by miR-24 and miR-101 did not interact with tetraspanin genes. Nevertheless, all genes analyzed are involved in the same processes. In particular, six proteins are involved in transportand regulation of cell motility and immune responses, and four proteins in endocytosis. It should be noted that 13 out of 14 proteins are involved in common pathways, with the exception of Tspan8.

**Figure 7:**
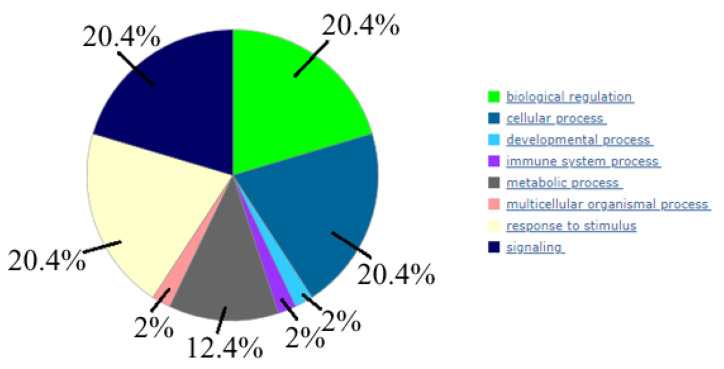
Biological processes, controlled by 14 genes (the following data is done using PANTHER (www.pantherb.org, (accessed on 30 May 2022)).

Then, 14 genes (ADCY3, ADCY6, ADCY7, IGF1R, MAP2K1, MAPK1, MAPK3, PRKACA, CD9, CD24, CD63, CD81, CD151, and TSPAN8) were analyzed using the PANTHER database to identify common pathways the encoded proteins were involved in; therefore, exploring which biological processes might be particularly over-represented in these gene groups (using the over-representation analysis) (Figure 7). 

We found that these genes predominantly involved such biological processes as “biological regulation”, “cellular process”, “response to stimulus”, and “signaling”.

Bioinformatics analysis indicated that the selected two tumor-associated miRNAs (miR-101 and miR-24-3p) and tetraspanins are involved in the regulation of key processes of OC carcinogenesis and are promising diagnostic markers and predictors of poor clinicopathological behavior in tumors.

## 3. Discussion

Although malignant cells themselves are the major source of tumorigenesis, their interactions with the tumor microenvironment are critical for the progression from a single tumor mass to distant metastases. Derived from cancer cells, exosomes mediate EMT, malignant cell proliferation and motility, metastatic processes, angiogenesis stimulation, and immune system repression [25,26,27]. It is known that exosomes contain cytoplasmic proteins and membrane proteins, functionally active ribo- (mRNA, miRNA, lncRNA, rRNA, tRNA, etc.) and deoxyribonucleic acids (genomic and mitochondrial DNA) [28,29]. Since the proportion of DNA in exosomes is negligible [6], it is assumed that RNA and protein cargo of cancer exosomes play the main role in recipient cells transformation. We are able to assume that ribonucleic acids (lncRNA and microRNA) and biologically active proteins should act synergistically on target cells. However, there are still very few researches devoted to the studying of the representation of tumor-associated RNAs and proteins and in exosomes simultaneously.

In the current study, we have chosen universal exosomal tetraspanins (CD9, CD63, and CD81) involved in exosome biogenesis, exosome protein sorting, cell adhesion, and exosome uptake by recipient cells [25,29,30], and less studied tetraspanins like CD151 and Tspan8 involved in extracellular matrix degradation, stromal remodeling, angiogenesis, and cell motility [7]. According to the Exocarta database (www.exocarta.org, accessed on 30 October 2022), exosomes are involved in the transport of 2838 microRNAs, the composition of which in microvesicles depends on the composition of parental cells and the general state of the organism. We have chosen miR-101 and miR-24-3p as microRNAs relevant for OC [18]. In particular, the overexpression of miR-101 can remarkably reduce the in vitro proliferation and invasion ability of OC cells through the down-regulation of SOCS-2, which is a major part of cytokine signaling suppressors and is involved in a series of important processes associated with cell growth, division, and apoptosis [31]. It was also found that the transcription factors ZEB1 and ZEB2 are targets for miR-101 were, which directly suppress the E-cadherin genes, and thus miR-101 suppresses EMT, cell migration, and invasion [19,32]. The low expression of this miRNA is characteristic of OC [33] and correlates with poor tumor differentiation and resistance to cisplatin [34]. It should be noted that tetraspanin CD151 contributes to survival of a subset of high-grade serous ovarian cancer cell lines associated with a ZEB transcriptional program and supports the growth of ovarian tumors as well [10]. As a result, we suggested indirect association between miR-101 and CD151 in OC progression.

MiR-24-3p plays an important role in migration, invasion, and proliferation of human carcinoma too [35]. For the first time, seven years ago, enriched analysis was used to identify potential functional associations of miRNA-disease (MDA) in humans, by integrating currently known biological data (miRNA-target interactions, protein-protein interactions, and gene-disease associations), resulting in the discovery of a new MDA, between miR-24 and OC. Compared to scramble miRNA, overexpression of miR-24 was used to remarkably induce OC cells apoptosis [36]. Then, using bioinformatics analysis, it was revealed that miR-24-3p contributes to ovarian cancer cell chemoresistance to cisplatin by mediating OVCAR-8R cell cisplatin resistance-related gene modules, which participated in functions that were closely related to “apoptosis”, “cell cycle”, and “adhesion” [20]. In the current year, it was found that miR-24-3p could suppress cancer metastasis through EMT in OC cells [37]. Despite intensive studies on the role of miRNA-24 in cancer pathways and the study of its level of expression in exosomes, there is no information in the literature on the possibility of the transfer of this miRNA by exosomes of plasma and ascites in OC. Since miR-24 is a CD44 target, and Tspan8 expression on the surface of some tumor cell cultures is mediated by CD44 [38], we also suggested an indirect relationship between miR-24 and Tspan8 in OC progression.

In this study, we investigated the alteration of the relative expression levels of tumor-associated microRNAs miR-24-3p and miR-101 in exosomes of plasma and ascites fluid of OC patients, because the alterations of miRNA expression in different biofluids may reflect different pathophysiological processes taking place at a particular moment in the tumor process. Samples of HFs were used as reference. Not surprisingly, the relative level of these microRNAs in the plasma exosomes of cancer patients was significantly lower than in healthy women and correlated with the level in ascites exosomes. It was revealed that unidirectional changes in the expression of these microRNAs associated with EMT and cell migration, stimulate cell proliferation and angiogenesis, indicating that miR-24-3p and miR-101 can be effective biomarkers of liquid biopsies and targets for successful anticancer therapy. Moreover, strong (on the Chaddock scale) direct correlation between the change in the ascites exosomes CD151+Tspan8+ subpopulation level and the ascites exosomal miR-24 expression level (R = 0.81, *p* < 0.05) in OC patients confirms assumption that exosomal cargo act synergistically to increase cellular motility, affecting cellular processes and signaling. In addition, the results indirectly indicate that, despite the low representation of the CD151+Tspan8+ subpopulation (about 3%) of exosomes, it is they that contribute to the transfer of signals from cancer cells to recipient cells and thus, to tumor dissemination itself. We revealed a strong significant correlation between the level of CD151+Tspan8+-ascitic CD9-positive exosomes and the relative level of miR-24-3p in the plasma exosomes of patients with OC, which brings us closer to the successful development of a non-invasive liquid biopsy, based on the analysis of the content of plasma exosomes of OC patients, since blood is a more accessible biofluid than ascites. Confirmed by bioinformatics analysis, involvement of CD151,Tspan8 tetraspanins, and genes controlled by miR-24-3p and miR-101 in signaling pathways, which are crucial for carcinogenesis, demonstrates that these tetraspanins and microRNAs are potential biomarkers in assessing the prognosis of metastasis and tumor drug resistance, as well as to select new targets for targeted therapy. Despite the encouraging data, further research in large groups of patients is needed to verify the association between CD151/Tspan8 and miR-24/miR-101 in OC. In addition, it remains to be seen whether the identified protein-microRNA patterns of exosomal cargo are universal for other types of cancer.

## 4. Materials and Methods

### 4.1. Female Enrollment

Blood samples from HFs (*n* = 19, median age 43) were obtained from Novosibirsk Central Clinical Hospital. HFs did not have any female disorders (dysplasia, endometriosis, etc.) or any malignant diseases, as well as cancer-related genetics.

Blood and ascites samples of previously untreated advanced OC patients (*n* = 20, median age 56 years) were obtained from the Department of Gynecology of the Cancer Research Institute of Tomsk National Research Medical Center (Table 2).

Blood samples from all enrolled patients were collected before therapy. The volume of ascites was measured clinically and by ultrasound examination. Three clinical subgroups of OC patients were formed: patients having low-volume (<200 mL), moderate-volume (200–1000 mL), and high-volume ascites (>1000 mL). The peritoneal carcinomatosis index was determined in all OC patients by thoracoabdominal computed tomography and/or preoperative laparoscopy. In the patients included in the study, the PCI varied from 8 to 16.

### 4.2. Isolation and Characterization of Exosomes

Exosomes from blood plasma (from 18 mL of venous blood) and ascites (about 18–20 mL) were isolated using ultrafiltration with ultracentrifugation as described previously [39]. Exosome samples (in 400 µL of PBS) from plasma and ascites fluid were aliquoted and stored either at −80 °C or in liquid nitrogen. The aliquots were thawed once before use.

Morphology of the isolated EVs from plasma and ascites was assessed by TEM as described previously [6]. The volumes of exosomes from plasma or ascites for the study of EV using the TEM were 15 µL.

To evaluate the exosomal protein concentration, a NanoOrange Protein Quantitation kit (NanoOrange^®^ Protein Quantitation Kit, Molecular Probes, Eugene, OR, USA) was used in accordance with the manufacturer’s recommendations. The volumes of exosomes from plasma or ascites for protein quantification were 8 µL.

Analysis of CD9/CD63/CD81/CD24 subpopulations in plasma and ascites fluid exosomes was carried out using flow cytometry as described previously [8]. The volumes of exosomes from plasma or ascites for flow cytometry were about 130 µL (30 μg exosomal protein). The MFI of stained exosomes was analyzed and compared to the isotype (BD bioscience, Heidelberg, Germany) and negative controls.

### 4.3. Analysis of Tspan8/CD151 Subpopulations on the Surface of CD9-Positive Exosomes

Aliquots of exosomes (about 30 μg vesicular protein) were incubated with 3 × 10^5^ 4 μm-diameter aldehyde/sulphate latex beads (Thermo Fisher Scientific, USA) labeled anti-CD9 antibody (ab134375, Abcam) in 150 μL of PBS at 4 °C overnight at gentle agitation and blocked in 0.2 M glycine for 30 min. The exosomes-antibody-bead complexes were washed twice with a washing buffer (2% EVs depleted bovine serum in PBS) and stained with anti-Tspan8-PE antibody (3 μL on test, ABIN4895321, Antibodies-online, Germany) and anti-CD151-APC antibody (3 μL on test, #350405, Biolegend, San-Diego, CA, USA) for 20 min at room temperature. All complexes were washed twice, suspended in 300 µL of FACS buffer and analyzed by flow cytometry on cytometer Cytoflex (Becman Coulter, Malaysia). Data were analyzed with CytExpert 2 Software. Single beads were gated, and tetraspanin compositions (%) at the surface of exosomes were calculated.

### 4.4. Bioinformatic Analysis

The participation of microRNAs (miR-101 and miR-24-3p) in the dissemination of OC was analyzed using the DIANA database, which contains information about microRNA target genes, as well as the processes and pathways in which they participate (https://dianalab.ece.uth.gr, accessed on 30 May 2022). Profiling of exosomal miR-101 and miR-24-3p and tetraspanins was performed using STRING software (https://string-db.org, accessed on 30 May 2022). In a finish bioinformatic analysis PANTHER Classification System was used (www.pantherdb.org, accessed on 30 May 2022).

### 4.5. Evaluation of miRNA Concentrations

Before the isolation of miRNA, samples of exosomes were thawed on ice and gently mixed. RNA was isolated from 66 µL of plasma exosomes or ascitic exosomes (obtained from 3 mL of blood or acsites for the analysis of three miRNAs) using miRNA Isolation Kit (Biosilica, Russia) according to the manufacturer’s instructions, precipitated with glycogen and isopropanol, and reconstituted in 35 μL of water [39]. After the addition of denaturation buffer, synthetic cel-miR-39-3p was spiked-in the samples at 5 × 10^7^ copies per isolation. The purity of isolated miRNA was determined by OD260/280, using a Nanodrop ND-1000 (Thermo Scientific, Wilmington, DE, USA). The quality of exosomal RNAs was evaluated using an “High Sensitivity RNA Kit” and an Agilent 2100 Bioanalyser^TM^ (Agilent Technologies, Waldbronn, Germany), in SB RAS Genomics Core Facility (ICBFM SB RAS, Russia).

Reverse transcription on miRNA templates was performed as described by Konoshenko et al. [40]. Primers and probes for reverse transcription and TaqMan qPCR (view Supplementary Materials, Table S4) were synthesized in the Laboratory of Medicinal Chemistry (ICBFM SB RAS, Novosibirsk). Each reverse transcription reaction was performed in a total volume of 10 µL and contained 4.5 µL of RNA, 25 nM each of miRNA-specific primers, 0.5 unit of RiboLock RNAse inhibitor (Fermentas, Vilnius, Lithuania), 50 units of M-MuLV-RH reverse transcriptase (Fermentas, Vilnius, Lithuania), 2.5 µL of 5× MMLV reaction buffer [250 mM Tris-HCl (pH 8.3 at 25 °C), 250 mM KCl, 20 mM MgCl_2_, 50 mM DTT], and 125 mM of each dNTP. The reaction conditions were as follows: 16°C for 30 min, 42 °C for 30 min, and 70 °C for 10 min. Samples without RNA templates were used as negative controls.

Real-time PCR was carried out on the LightCycler 480 II detection system (Roche, Switzerland). All reactions were carried out in duplicate in a total volume of 49 µL. Each reaction contained 9 µL of reverse transcription product, 1 unit of Taq DNA polymerase (Fermentas, Vilnius, Lithuania), 2.4 µL of 10× PCR buffer [750 mM TrisHCl (pH 8.8 at 25 °C), 200 mM (NH_4_)_2_SO_4_, 0.1% (*v*/*v*) Tween 20], 3.2 mM MgCl_2_, 200 mM of each dNTP, 480 nM miRNA-specific forward primer, 640 nM universal reverse primer, and 240 nM specific TaqMan probe (see Supplementary Materials, Table S4). After an initial denaturation at 95 °C for 3 min, the reactions were run for 45 cycles at 95 °C for 15 s and 60 °C for 45 s. The relative expression values of target miRNAs were normalized to miR-16 and were calculated following the ddCt method, as described previously [41].

### 4.6. Statistics

Statistical analysis was performed using Statistica 10.0 software. Each data set was first tested for a Gaussian distribution by using a Shapiro–Wilk normality test. All data were expressed as medians with interquartile ranges or as means with standard errors. To evaluate the difference, Mann–Whitney U test and Kruskal–Wallis test were used. Correlation analysis on data was carried out with Spearman’s Rank Correlation test. *p*-values < 0.05 were considered to be statistically significant.

## Figures and Tables

**Table 1:** Tetraspanin composition (%) at the surface of exosomes in HF and OC patient samples, Medians (25–75%).

Subpopulations	HFs (*n* = 19)	OC Patients (*n* = 20)	*p*-Level
*Plasma exosomes*			
Tspan8+	6.9 (5.47–8.96)	11.02 (7.66–15.6)	*0.046*
CD151+	1.88 (1.63–2.82)	3.91 (2.99–5.02)	*0.011*
CD151+/Tspan8+	1.59 (1.31–2.29)	3.22 (2.76–4.80)	*0.006*
*Ascites exosomes*			
Tspan8+		7.31 (5.40–9.10)	
CD151+		3.40 (2.45–4.96)	
CD151+/Tspan8+		2.71 (1.77–3.82)	

**Table 2:** General clinical characteristics of OC patients.

	OC Patients, *n* = 20
Histology	
Serous	20 (100%)
FIGO (2013) staging	
IIB	2 (10%)
IIIB	3 (15%)
IIIC	15 (75%)
Tumor Grade	
High-grade	20 (100%)
Ascites volume	
<200 mL	5 (25%)
200–1000 mL	5 (25%)
>1000 mL	10 (50%)

## Supplementary Materials

The following supporting information can be downloaded at: https://www.mdpi.com/article/10.3390/ijms24010464/s1.
