# Supplementary material for: Comparative Analysis of Tumor-Associated microRNAs and Tetraspanines from Exosomes of Plasma and Ascitic Fluids of Ovarian Cancer Patients
Source: Int J Mol Sci. 2022 Dec 27;24(1):464. doi: 10.3390/ijms24010464 (PMC9820379; doi:10.3390/ijms24010464)
Supplement: Supplementary file 1 [file ijms-24-00464-s001.zip › ijms-2052366-supplementary.pdf]

Table S1. Expression of CD63, CD81 and CD24 on the surface of CD9-positive exosomes from blood plasma (HFs, OCPs) and ascites (OCPs).

Source of exosomes		CD63 expression	CD81 expression	CD24 expression
HFs	Plasma	530 [490; 600]	715 [630;850]	960 [750; 1100]
OCPs	Plasma	850 [700; 950]	950 [880; 1200]	1350 [1050; 1500]
	Ascites	890 [800; 1200]	950 [880; 1230]	2300 [2000; 2555]
		p=0.005	p=0.056	p=0.004

Note: Data represented median fluorescence intensity (MFI) with 25-75%. Kruskal-Wallis test was used.

Table S2. The level of exosomal miR-101 in plasma and ascites in patients with OC in relation to clinical parameters, Medians (27-75%).

Parameters, n	Plasma miR-101 level	P-level	Ascites miR-101 level	P-level
**Age (years),**				
<56, n = 10	7.03 [6.35; 7.71]	0.89	6.77 [5.70; 7.20]	0.42
≥56, n = 10	7.27 [6.13; 7.78]		6.93 [6.41; 8.05]	
**FIGO Stage**				
IIB-IIIAB, n = 5	7.25 [5.80; 7.75]	0.94	**5.41 [3.49; 6.09]**	**0.03**
IIIC, n = 15	6.99 [6.35; 7.60]		**7.05 [6.50; 7.40]**	
**Family history**,				
Yes, n = 4	6.67 [5.73; 7.61]	0.63	7.12 [7.04; 7.20]	0.63
No, n = 16	7.21 [6.35; 7.23]		6.65 [5.90; 7.53]	
**Ascites volume**				
**(mL**),	6.47 [6.04; 6.89]		6.10 [5.50; 6.77]	
<200 mL, n = 5	6.86 [6.13; 7.78]	0.53	7.62 [6.65; 8.62]	0.14
200-1000 mL, n = 5	7.57 [5.80; 7.71]		7.20 [6.30; 7.40]	
>1000ml, n = 10				
**PCI, RU**				
≤9, n = 10	6.98 [6.35; 7.75]	0.80	6.65 [5.70; 8.00]	0.44
>9, n = 10	7.18 [6.04; 7.66]		7.12 [6.77; 7,30]	

Note: RU – Relative Units, PCI – peritoneal canceromatosis index

Table S3. The level of exosomal miR-24 in plasma and ascites in patients with OC in relation to clinical parameters, Medians (27-75%).

Parameters, n	Plasma miR-24 level	P-level	Ascites miR-24 level	P-level
**Age (years),**				
<56, n = 10	-6.45 [-7.40; -5.75]	0.67	-5.70 [-8.61; -3.64]	0.75
≥56, n = 10	-6.39 [-9.10; -4.80]		-5.55 [-9.00; -5.00]	
**FIGO Stage**				
IIB-IIIAB, n = 5	-6.86 [-8.00; -0.80]	0.89	-3.77 [-10.80; -2.24]	0.78
IIIC, n = 15	-6.39 [-7.59; -5.75]		-5.70 [-8.80 ;-5.00]	
**Family history,**				
Yes, n = 4	-5.75 [-8.00; -5.20]	1.00	-3.64 [-4.48; -3.41]	1.00
No, n = 16	-6.45 [-8.00; -4.80]		-5.70 [-9.00; -3.77]	
**Ascites volume**				
**(mL),**	-6.00 [-6.30 ;-5.75]		-5.50 [-5.70; -3.64]	
<200 mL, n = 5	-7.91 [-8.66; -6.20]	0.35	-8.90 [-9.55; -5.61]	0.43
200-1000 mL, n = 5	-6.45 [-7.40; -4.30]		-5.33 [-8.61; -3.77]	

>1000ml, n = 10				
**PCI, RU**				
≤9, n = 10	-7.80 [-8.22; -6.00]	0.35	-8,90 [-10.11; -5.70]	0.22
>9, n = 10	-6.39 [-7.40; -4.80]		-5.33 [-8.61; -3.64]	

Note: RU – Relative Units, PCI – peritoneal canceromatosis index

Table S4. Sequences of primers and probes used for reverse transcription and TaqMan qPCR.

miRNA	Label	Sequence
Universal Reverse Primer		5′-GTGCAGGGTCCGAGGT-3′
hsa-miR-16-5p (miR-16)	RT	5′-GTCGTATCCAGTGCAGGGTCCGAGGTATTCGCACTGGATACGACCGCCAA-3′
	Reverse	5′-GCCCGTAGCAGCACGTAAATAT-3′
	Probe	5′-(FAM)-GCACTGGATACGACCGCCAA-(BHQ1)-3′
hsa-miR-24-3p (miR-24-3p)	RT	5′-GTCGTATCCAGTGCAGGGTCCGAGGTATTCGCACTGGATACGACCTGTTC-3′
	Reverse	5′-TGGCTCAGTTCAGCAG-3′
	Probe	5′-(FAM)-CGCACTGGATACGACCTGTTC-(BHQ1)-3′
hsa-mir-101 (miR-101)	RT	5′-GTCGTATCCAGTGCAGGGTCCGAGGTATTCGCACTGGATACGACTTCAGT-3′
	Reverse	5′-CGCCC TACAGTACTGTGATAA-3′
	Probe	5′-(FAM)-CGCACTGGATACGACTTCAGT-(BHQ1)-3′
